# Etiology and antibacterial susceptibility pattern of community-acquired bacterial ocular infections in a tertiary eye care hospital in south India

**DOI:** 10.4103/0301-4738.71678

**Published:** 2010

**Authors:** M Jayahar Bharathi, R Ramakrishnan, C Shivakumar, R Meenakshi, D Lionalraj

**Affiliations:** Department of Microbiology, India; Chief Medical Officer, Aravind Eye Hospital & Postgraduate Institute of Ophthalmology, Tirunelveli, Tamil Nadu, India; General Ophthalmology, Aravind Eye Hospital & Postgraduate Institute of Ophthalmology, Tirunelveli, Tamil Nadu, India; Cornea & Refractive Surgery, Aravind Eye Hospital & Postgraduate Institute of Ophthalmology, Tirunelveli, Tamil Nadu, India

**Keywords:** Antibacterial agents, bacterial pathogens, etiology, *in vitro* susceptibilities, ocular infection

## Abstract

**Aims:**

To identify the etiology, incidence and prevalence of ocular bacterial infections, and to assess the in vitro susceptibility of these ocular bacterial isolates to commonly used antibiotics.

**Materials and Methods:**

Retrospective analysis of consecutive samples submitted for microbiological evaluation from patients who were clinically diagnosed with ocular infections and were treated at a tertiary eye care referral center in South India between January 2002 and December 2007.

**Results:**

A total of 4417 ocular samples was submitted for microbiological evaluation, of which 2599 (58.8%) had bacterial growth, 456 (10.3%) had fungal growth, 15 (0.34%) had acanthamoebic growth, 14 (0.32%) had mixed microbial growth and the remaining 1333 (30.2%) had negative growth. The rate of culture-positivity was found to be 88% (*P* < 0.001) in eyelids’ infection, 70% in conjunctival, 69% in lacrimal apparatus, 67.4% in corneal, 51.6% in intraocular tissues, 42.9% in orbital and 39.2% in scleral infections. The most common bacterial species isolated were *Staphylococcus aureus* (26.69%) followed by *Streptococcus pneumoniae* (22.14%). *Sta. aureus* was more prevalent more in eyelid infections (51.22%; *P* = 0.001) coagulase-negative staphylococci in endophthalmitis (53.1%; *P* = 0.001), Str. pneumoniae in lacrimal apparatus and corneal infections (64.19%; *P* = 0.001), *Corynebacterium* species in blepharitis and conjunctivitis (71%; *P* = 0.001), *Pseudomonas aeruginosa* in keratitis and dacryocystitis (66.5%; *P* = 0.001), *Haemophilus* species in dacryocystitis and conjunctivitis (66.7%; *P* = 0.001), *Moraxella lacunata* in blepharitis (54.17%; *P* = 0.001) and *Moraxella catarrhalis* in dacryocystitis (63.83%; *P* = 0.001). The largest number of gram-positive isolates was susceptible to moxifloxacin (98.7%) and vancomycin (97.9%), and gram-negative isolates to amikacin (93.5%) and gatifloxacin (92.7%).

**Conclusions:**

Gram-positive cocci were the most frequent bacteria isolated from ocular infections and were sensitive to moxifloxacin and vancomycin, while gram-negative isolates were more sensitive to amikacin and gatifloxacin.

The eye may be infected from external sources or through intraocular invasion of micro-organisms that are carried by the blood stream.[1] External bacterial infections of the eye are usually localized but may frequently spread to other tissues. The eyelid and conjunctiva have a normal microbial flora controlled by its own mechanism and by the host. Modification of this normal flora contributes to ocular infections such as blepharitis, conjunctivitis, canaliculitis, orbital cellulitis, endophthalmitis, etc.[1–5] Timely institution of appropriate therapy must be initiated to control the infections and thereby minimize ocular morbidity. If they are not treated promptly, it may lead to sight threatening condition. For specific antibacterial treatment, isolation and identification of bacterial pathogens along with antibiotic susceptibility spectrum is essential. The bacterial etiology and their susceptibility as well as resistance patterns may vary with geographic location according to the local population.[6,7] *Streptococcus pneumoniae* was reported to be the predominant corneal pathogen in Tiruchirapalli[8] and Madurai,[9] whereas in Coimbatore it was *Pseudomonas aeruginosa*.[10] *Ps. aeruginosa* was reported to be the most common bacterial pathogen causing postoperative endophthalmitis in Chennai,[11] whereas in Madurai it was *Nocardia sp*.[12] Similarly, there was a variation in the *in vitro* efficacy of antibacterial agents against bacterial pathogens causing ocular infections according to the local population. For instance, ciprofloxacin showed higher sensitivity against keratitis pathogens in Tirunelveli (90%)[13] than in Hyderabad (69.3%).[14] Thus, the current trends in the etiology of bacterial ocular infections and their susceptibilities must be updated to make a rational choice of initial antibiotic therapy. The purpose of this study was to identify the etiology, incidence and prevalence of ocular bacterial infections, and to assess the *in vitro* susceptibility of these ocular bacterial isolates to commonly used antibiotics.

## Materials and Methods

This retrospective, noncomparative and consecutive analysis included samples submitted for microbiological evaluation, from patients clinically diagnosed with ocular infections such as blepharitis, conjunctivitis, internal and external hordeolum, suppurative scleritis, canaliculitis, keratitis, dacryocystitis, preseptal cellulitis, endophthalmitis and panophthalmitis, and treated at a tertiary eye care referral center located at Tirunelveli district, Tamil Nadu, South India, between January 2002 and December 2007. All the patients were examined on the slit-lamp biomicroscope and infective diseases included in this study were diagnosed clinically by a group of ophthalmologists.[4,5]

After detailed ocular examinations using standard techniques,[15,16] specimens for culture and smear were obtained by scraping the eyelid margin using a sterile blade (#15) on a Bard-Parker handle and by swabbing the lid margins with sterile broth-moistened cotton swabs in cases of blepharitis. Similarly, specimens were also obtained from the corneal ulcers by scraping. For cases of suppurative scleritis, specimens were collected by scraping and swabbing the area of the suppurative abscess. Conjuctival cultures were obtained by wiping a broth-moistened swab across the lower conjunctival cul-de-sac in conjunctivitis cases, and thick, tenacious purulent punctal discharge was collected from the canaliculus by pressure applied over the area of the eyelid that overlies the canaliculus in cases of canaliculitis. In cases of external and internal hordeolum, the abscesses were incised and the drained pus was obtained. From the cases of dacryocystitis, purulent material was collected from everted punta by applying pressure over the lacrimal sac area, and from the surgically excised lacrimal sac. Specimens from cases of preseptal cellulitis were obtained after stab incision or through an open wound or drainage site, if present. In patients in whom infectious endophthalmitis and panophthalmitis are suspected, lid and conjunctival specimens along with anterior chamber and vitreous fluids were obtained.

The obtained extraocular and intraocular specimens were inoculated directly onto the blood agar (5% defibrinated sheep blood in tryptose blood agar base with yeast extract), chocolate agar, Sabouraud’s dextrose agar (Emmons modification), thioglycolate medium and brain-heart infusion broth, and specimens from lacrimal apparatus, cornea and vitreous were also inoculated onto Lowenstein-Jensen agar slant. In addition, all corneal scrapes were inoculated onto non-nutrient agar for Acanthamoeba culture. A part of the collected specimens was subjected to 10% KOH wet mounting, Gram’s and Giemsa’s staining procedures, and if needed, Kinyoun’s and Ziehl-Neelsen acid-fast staining procedures were also performed. A positive culture was defined as a growth of the same organisms on more than two solid phase media or confluent growth on one solid medium. A standardized protocol was followed for each ocular specimen for the evaluation of significant microbiological features.[15,16] *In vitro* susceptibility testing was performed by Kirby-Bauer disc diffusion method and interpreted using Clinical and Laboratory Standards Institute’s serum standards.[17] The antibacterial agents (Hi-media Laboratories Pvt. Ltd., Mumbai, India) used were amikacin (30 μg/disk), tobramycin (10 μg/disk), gentamicin (10 μg/disk), cefazolin (30 μg/disk), cephotaxime (30 μg/disk), ceftazidime (30 μg/disk), ciprofloxacin (5 μg/disk), norfloxacin (10 μg/disk), ofloxacin (5 μg/disk), gatifloxacin (5 μg/disk), moxifloxacin (5 μg/disk), chloramphenicol (30 μg/disk) and vancomycin (30 μg/disk) and were consistently tested for their efficacy against standard American Type Culture Collection (ATCC) bacteria (*Staphylococcus aureus* ATCC 25923, *Str. pneumoniae* ATCC 49619, *Haemophilus influenzae* ATCC 49241, *Ps. aeruginosa* ATCC 27853, *Escherichia coli* ATCC 25922) as a general quality control laboratory procedure.

Statistical software (STATA 8.1, Stata Corporation, Texas, USA) was used for statistical analysis. The statistical analysis was carried out to determine the difference in the rate of recovery of microbes from various ocular specimens and also to determine the significance in the prevalence of common bacterial species in causing ocular infections. Pearson’s chi-square test was used for analysis and a *P* value <0.05 was considered statistically significant.

## Results

A total of 4417 ocular samples obtained from 4372 patients clinically diagnosed with o cular infections, was submitted for microbiological evaluation during the study period of 6 years. Of the 4372 patients, single eye was infected in 4327 (98.97%) patients and both the eyes were infected in 45 (1.03%) patients; thus, a total of 4417 (4327 + 90) eyes with ocular infections was studied [Table 1]. Of the 4417 ocular specimens subjected to cultures, 2599 (58.8%) had bacterial growth, 456 (10.3%) had fungal growth, 15 (0.34%) had acanthamoebic growth and 14 (0.32%) had mixed microbial growth. The remaining 1333 (30.2%) ocular specimens were culture negative for microbial growth [Table 2]. The rate of culture-positivity was found to be significantly higher among eyes with eyelids’ infection (88%; 677 of 766) than eyes with conjunctival (69.7%; 576 of 826) (*P* = 0.001), lacrimal apparatus (69%; 729 of 1057) (*P* = 0.001), corneal (67.4%; 846 of 1256) (*P* = 0.001), intraocular tissues (51.6%; 227 of 440) (P = 0.001), orbital (42.9%; 9 of 21) (*P* = 0.001) and scleral (39.2%; 20 of 51) (*P* = 0.001) infections [Table 1].

**Table 1:** Culture results of ocular specimens obtained from eyes with ocular infection between 2002 and 2007 at a tertiary eye care center in South India

Name of the bacterial ocular infection	Total number of patients from whom ocular specimens were collected and submitted for culture and sensitivity test (%)	Number of patients from whom ocular specimens were collected from single eye (%)	Number of patients from whom ocular specimens were collected from both eyes (%)	Total number of ocular specimens collected and subjected to culture and sensitivity test (%)	Number of specimens collected from eyes, which showed positive culture (%)	Number of the specimens collected from eyes, which showed negative cultures (%)
Infections of the eyelids	751 (17.18)	736 (17.01)	15 (33.33)	766 (17.34)	677/766 (88.38)	89/766 (11.62)
Blepharitis	530	515	15	545 (515 + 30)	491/545 (90.09)	54/545 (9.91)
Hordeolum	190	190	0	190	171/190 (90)	19/190 (0.1)
Preseptal cellulitis	31	31	0	31	15/31 (48.39)	16/31 (51.61)
Infections of the conjunctiva	799 (18.28)	772 (17.84)	27 (60)	826 (18.7)	576/826 (69.73)	250/826 (30.27)
Conjunctivitis	775	748	27	802 (748 + 54)	560/802 (69.83)	242/802 (30.17)
Blebitis	24	24	0	24	16/24 (66.67)	8/24 (33.33)
Infections of the orbit	21 (0.48)	21 (0.49)	0	21 (0.47)	9/21 (42.86)	12/21 (57.14)
Orbital cellulites	21	21		21	9/21 (42.86)	12/ 21 (57.14)
Infections of the lacrimal apparatus	1057 (24.18)	1057 (24.43)	0	1057 (23.93)	729/1057 (68.97)	328/1057 (31.03)
Dacryocystitis	930	930		930	651/930 (70)	279/930 (30)
Lacrimal abscess	16	16		16	14/16 (87.5)	2/16 (12.5)
Canaliculus	111	111		111	64/111 (57.66)	47/111 (42.34)
Infections of the cornea	1253 (28.66)	1250 (28.89)	3 (6.67)	1256 (28.44)	846/1256 (67.36)	410/1256 (32.64)
Keratitis	1253	1250	3	1256 (1250 + 6)	846/1256 (67.36)	410/1256 (32.64)
Infections of the sclera	51 (1.17)	51 (1.18)	0	51 (1.15)	20/51 (39.22)	31/51 (60.78)
Scleritis	51	51		51	20/51 (39.22)	31/51 (60.78)
Infections of the intraocular tissues	440 (10.06)	440 (10.17)	0	440 (9.96)	227/440 (51.59)	213/440 (48.41)
Postoperative endophthalmitis	307	307		307	110/307 (35.83)	197/307 (64.17)
Post-traumatic endophthalmitis	93	93		93	89/93 (95.7)	4/93 (4.3)
Endogenous endophthalmitis	13	13		13	9/13 (69.23)	4/13 (30.77)
Post-corneal endophthalmitis	11	11		11	11/11 (100)	0/11 (0)
Post-scleral endophthalmitis	7	7		7	3/7 (42.86)	4/7 (57.14)
Panophthalmitis	9	9		9	5/9 (55.56)	4/9 (44.44)
Total number (%)	4372 (100)	4327/4372 (98.97)	45/4372 (1.03)	4417 (100)	3084/4417 (69.82)	1333/4417 (30.18)

**Table 2:** Microbial growth pattern of ocular specimens obtained from eyes (*n* = 4417) with ocular infections subjected to culture and sensitivity test between 2002 and 2007 at a tertiary eye care referral centre in South India

Name of the bacterial ocular infection	Total number of ocular specimens collected and subjected to culture and sensitivity test (%)	Eyes that showed positive microbial growth					Number of specimens collected from eyes, which showed negative cultures (%)
		Number of specimens collected from eyes, which showed positive culture (%)	Number of specimens (eyes) with bacterial growth alone (%)	Number of specimens (eyes) with fungal growth alone (%)	Number of specimens (eyes) with acanthamoebic growth alone (%)	Number of specimens (eyes) with mixed microbial growth (%)
Infections of the eyelids	766 (17.34)	677 (88.38)	677 (88.38)	0	0	0	89 (11.62)
Blepharitis	545 (515 + 30)	491 (90.09)	491 (90.09)				54 (9.91)
Hordeolum	190	171 (90)	171 (90)				19 (0.1)
Preseptal cellulites	31	15 (48.39)	15 (48.39)				16 (51.61)
Infections of the conjunctiva	826 (18.7)	576 (69.73)	576 (69.73)	0	0	0	250 (30.27)
Conjunctivitis	802 (748 + 54)	560 (69.83)	560 (69.83)				242 (30.17)
Blebitis	24	16 (66.67)	16 (66.67)				8 (33.33)
Infections of the orbit	21 (0.47)	9 (42.86)	9 (42.86)	0	0	0	12 (57.14)
Orbital cellulitis	21	9 (42.86)	9 (42.86)				12 (57.14)
Infections of the lacrimal apparatus	1057 (23.93)	729 (68.97)	729 (68.97)	0	0	0	328 (31.03)
Dacryocystitis	930	651 (70)	651 (70)				279 (30)
Lacrimal abscess	16	14 (87.5)	14 (87.5)				2 (12.5)
Canaliculitis	111	64 (57.66)	64 (57.66)				47 (42.34)
Infections of the cornea	1256 (28.44)	846 (67.36)	412 (32.8)	405 (32.2)	15 (1.19)	14 (11.11)	410 (32.64)
Keratitis	1256 (1250 + 6)	846 (67.36)	412 (32.8)	405 (32.2)	15 (1.19)	14 (11.11)	410 (32.64)
Infections of the sclera	51 (1.15)	20 (39.22)	20 (39.22)	0	0	0	31 (60.78)
Scleritis	51	20 (39.22)	20 (39.22)				31 (60.78)
Infections of the intraocular tissues	440 (9.96)	227 (51.59)	176 (40)	51	0	0	213/440 (48.41)
Postoperative endophthalmitis	307	110 (35.83)	104 (33.88)	6			197/307 (64.17)
Post-traumatic endophthalmitis	93	89 (95.7)	44 (47.31)	45			4/93 (4.3)
Endogenous endophthalmitis	13	9 (69.23)	9 (69.23)				4/13 (30.77)
Post-corneal endophthalmitis	11	11 (100)	11 (100)				0/11 (0)
Post-scleral endophthalmitis	7	3 (42.86)	3 (42.86)				4/7 (57.14)
Panophthalmitis	9	5 (55.56)	5 (55.56)				4/9 (44.44)
Total (%)	4417 (100)	3084 (69.82)	2599 (58.8)	456 (10.3)	15 (0.34)	14 (0.32)	1333 (30.18)

Of 2599 eyes with bacterial growth alone, 2587 (99.54%) had infection with single species of bacteria and the remaining 12 (0.46%) had infection with two species of bacteria, and thus, a total of 2611 (2587 + 24) bacterial isolates was recovered [Table 3]. The predominant bacterial species isolated was *Sta. aureus* (26.69%; 697 of 2611), followed by *Str. pneumoniae* (22.14%; 578 of 2611), *Ps. aeruginosa* (8.35%; 218 of 2611), *Corynebacterium species* (7.93%; 207 of 2611), *Haemophilus* species (5.86%; 153 of 2611) and *Moraxella catarrhalis* (3.6%; 94 of 2611) [Table 4].

**Table 3:** Bacterial growth pattern of ocular specimens obtained from eyes (*n* = 2599) with ocular infections subjected to culture and sensitivity test between 2002 and 2007 at a tertiary eye care referral center in South India

Name of the bacterial ocular infection	Total number of specimens (eyes) with bacterial growth alone (%)	Number of specimens (eyes) with single species of bacterial growth (%)	Number of specimens (eyes) with two species of bacterial isolates (%)	Total number of bacterial isolates recovered (no. of pure isolates + no. of bacterial isolates mixed with other bacteria)
Infections of the eyelids	677 (26.05)	675/677 (99.7)	2/677 (0.3)	679 (675 + 4)
Blepharitis	491	489	2	493 (489 + 4)
Hordeolum	171	171	0	171 (171 + 0)
Preseptal cellulitis	15	15	0	15 (15 + 0)
Infections of the conjunctiva	576 (22.16)	576/576 (100)	0	576 (576 + 0)
Conjunctivitis	560	560	0	560 (560 + 0)
Blebitis	16	16	0	16 (16 + 0)
Infections of the orbit	9 (0.35)	9/9 (100)	0	9 (9 + 0)
Orbital cellulitis	9	9	0	9 (9 + 0)
Infections of the lacrimal apparatus	729 (28.05)	725/729 (99.45)	4/729 (0.55)	733 (725 + 8)
Dacryocystitis	651	648	3	654 (648 + 6)
Lacrimal abscess	14	14	0	14 (14 + 0)
Canaliculitis	64	63	1	65 (63 + 2)
Infections of the cornea	412 (15.85)	408/412 (99.03)	4/412 (0.97)	416 (408 + 8)
Keratitis	412	408	4	416 (408 + 8)
Infections of the sclera	20 (0.77)	20/20 (100)	0	20 (20 + 0)
Scleritis	20	20	0	20 (20 + 0)
Infections of the intraocular tissues	176 (6.77)	174/176 (98.86)	2/176 (1.14)	178 (174 + 4)
Postoperative endophthalmitis	104	104	0	104 (104 + 0)
Post-traumatic endophthalmitis	44	43	1	45 (43 + 2)
Endogenous endophthalmitis	9	9	0	9 (9 + 0)
Post-corneal endophthalmitis	11	10	1	12 (10 + 2)
Post-scleral endophthalmitis	3	3	0	3 (3 + 0)
Panophthalmitis	5	5	0	5 (5 + 0)
Total	2599 (100)	2587/2599 (99.54)	12/2599 (0.46)	2611 (2587 + 24)

**Table 4:** Bacterial pathogens recovered from ocular specimens obtained from (*n* = 2599) eyes with bacterial infections

Name of the bacterial isolate	Total no. of the bacterial isolates recovered (%)	Number of pure isolates (%)	Mixed with other bacterial isolates (%)
Total gram-positive cocci	1553 (59.48)	1541/1553 (99.23)	12/1553 (0.77)
*Sta. aureus*	697	695	2
*CoNS*	162	160	2
*Str. pneumoniae*	578	571	7
*Streptococcus pyogenes*	42	42	0
*Streptococcus viridans*	74	73	1
Total gram-positive bacilli	234 (8.96)	227/234 (97.01)	7/234 (2.99)
*Bacillus* spp.	27	26	1
*Corynebacterium* spp.	207	201	6
Gram-negative cocci	169 (6.47)	169/169 (100)	0
*M. lacunata*	48	48	0
*M. catarrhalis*	94	94	0
*Neisseria gonorrhea*	11	11	0
*Neisseria meningitidis*	3	3	0
*Acinetobacter calcoaceticus*	13	13	0
Aerobic actinomycetes	53 (2.03)	51/53 (96.23)	2/53 (3.77)
*No. asteroides*	53	51	2
Gram-negative bacilli	602 (23.06)	599/602 (99.5)	3/602 (0.5)
*Ps. aeruginosa*	218	215	3
*Es. coli*	24	24	0
*H. influenzae*	90	90	0
*Haemophilus parainfluenzae*	48	48	0
*Haemophilus aegypticus*	15	15	0
*Enterobacter agglomerans*	16	16	0
*Enterobacter aerogenes*	49	49	0
*Enterobacter cloacae*	5	5	0
*Klebsiella ozaenae*	9	9	0
*Klebsiella pneumoniae*	76	76	0
*Citrobacter diversus*	10	10	0
*Citrobacter freundic*	7	7	0
*Alcaligenes faecalis*	9	9	0
*Proteus mirabilis*	11	11	0
*Proteus vulgaris*	5	5	0
*Serratia marcesens*	10	10	0
Total	2611 (100)	2587/2611 (99.08)	24/2611 (0.92)

The predominant bacterial species isolated from eyes with blepharitis (46.7%; 230 of 493 total blepharitis bacterial isolates), hordeolum (71.9%; 123 of 171 total hordeolum bacterial isolates), preseptal cellulitis (26.7%; 4 of 15 total preseptal cellulitis bacterial isolates), conjunctivitis (41.3%; 231 of 560 total conjunctivitis bacterial isolates) and canaliculitis (33.8%; 22 of 65 total canaliculitis isolates) was Sta. aureus. From cases of dacryocystitis (31.4%; 205 of 654 total dacryocystitis isolates) and keratitis (37.02%; 154 of 416 total keratitis isolates) *Str. pneumoniae* was isolated, and from cases of postoperative (68.27%; 71 of 104 total postoperative endophthalmitis isolates) and post-traumatic endophthalmitis (37.5%; 15 of 45 total post-traumatic endophthalmitis isolates), coagulase negative staphylococci (CoNS) were predominantly recovered. Of 12 post-corneal infective endophthalmitis, 6 (50%) were found to be due to *Ps. aeruginosa* growth [Table 5].

**Table 5:** Association between the bacterial pathogens recovered from ocular specimens (*n* = 2599) and eyes with ocular infections treated at a tertiary eye care referral center in South India

Name of the bacterial isolates recovered	No. of bacterial isolates recovered^a^	Eyelid infections			Conjunctiva		Orbit	Lacrimal apparatus			Cornea	Sclera	Intraocular tissues					Panoph-thalmitis
		Blepharitis	Hordeolum	Preseptal cellulitis	Conjunctivitis	Blebitis	Orbital cellulitis	Dacryocystitis	Lacrimal abscess	Canaliculus	Keratitis	Scleritis	Postoperative endophthalmitis	Posttraumatic endophthalmitis	Endogenous endophthalmitis	Postcorneal endophthalmitis	Postscleral endophthalmitis	
Total gram-positive cocci	1553 (12)	332 (2)	165	9	336	10	6	325 (4)	11	38	197 (5)	6	86	22	2	4(1)	1	3	
*Sta. aureus*	697 (2)	230 (1)	123	4	231	2	3	70(1)	2	22	4	2	2	0	1	0	0	1	
CoNS	162 (2)	13	12	0	6	1	0	9	0	4	31 (2)	0	71	15	0	0	0		
*Str. pneumoniae*	578 (7)	65(1)	24	1	91	0	1	205 (2)	3	9	154 (3)	2	12	4	1	4(1)	1	1	
*Streptococcus pyogenes*	42	6	6	4	2	0	2	9	6	2	2	2	0	0				1	
*Streptococcus viridans*	74(1)	18	0	0	6	7	0	32(1)		1	6	0	1	3					
Total gram-positive bacilli	234 (7)	80 (2)	6	1	84	0		3(1)	0	16(1)	27(1)	3	2	12(2)					
*Bacillus* spp.	27(1)	6	0	1	11			0		0	2	0	0	7(1)					
*Corynebacterium* spp.	207 (6)	74 (2)	6	0	73			3(1)		16(1)	25(1)	3	2	5(1)					
Gram-negative cocci	169 (0)	35 (0)		1	38	0		65	0		25	4	0		1				
*M. lacunata*	48	26			11			0			11								
*Ne. gonorrhea*	11	2			6			0			3								
*Ne. meningitides*	3	0			2			0			0				1				
*M. catarrhalis*	94	6		1	15			60			9	3							
*Ac. calcoaceticus*	13	1			4			5			2	1							
Aerobic actinomycetes	53 (2)	0			0	0		0		11 (1)	26(1)	2	5	6		2	1		
*No. asteroides*	53 (2)	0			0			0		11 (1)	26(1)	2	5	6		2	1		
*Gram-negative bacilli*	602 (3)	46 (0)		4	102	6	3	261 (1)	3		141 (1)	5	11	5	6	6(1)	1	2	
*Ps. aeruginosa*	218	21		2	26		1	69(1)	3		76(1)	2	6	2	2	6(1)	1	1	
*Es. coli*	24	0		0	4		0	11			6	0	2	1	0				
*H. influenzae*	90	7		1	22	6	1	38			9	3	0		2			1	
*H. parainfluenzae*	48	3		0	11		0	29			4		0		1				
*H. aegypticus*	15	0		1	6		1	6			1		0						
*En. agglomerans*	16	2			2			8			4		0						
*En. aerogenes*	49	5			9			14			19		2						
*En. cloacae*	5	0			1			2		2		0					
*K.. ozaenae*	9	0			2			6		1		0					
*K. pneumoniae*	76	6			11			47		9		1	1	1			
*Ci. diversus*	10	0			1			6		2			1				
*Ci. freundic*	7	2			0			4		1							
*A.I. faecalis*	9	0			1			6		2							
*Pr. mirabilis*	11	0			2			7		2							
*Pr. vulgaris*	5	0			0			4		1							
*Se. marcesens*	10	0			4			4		2							
Total	2611 (24)	493 (4)	171	15	560	16	9	654 (6)	14	65(2)	416(8)	20	104	45 (2)	9	12(2)	3	5

* Total number of isolates: number of single species of bacteria + number of two species of bacteria (number of two species of bacteria)

Significantly more number of *Sta. aureus* was recovered from eyes with eyelid infections (51.22%; 357 of 697 total *Sta. aureus* isolates) than from eyes with other ocular infections (48.79%; 340 of 697) (*P* = 0.001). CoNS were recovered more from eyes with endophthalmitis (53.1%; 86 of 162) than from eyes with any other ocular infections (46.9%; 76 of 162) (*P* = 0.001). *Str. pneumoniae* was recovered significantly from more number of eyes with lacrimal apparatus infections and corneal infections [64.19%; 371 (217 from lacrimal apparatus and 154 from corneal infection) of 578 of total *Str. pneumoniae* isolates] than any other ocular infections (35.8%; 207 of 578) (*P* = 0.001). *Streptococcus viridans* was isolated frequently from eyes with dacryocystitis (43.24%; 32 of 74 total *Str. viridans* isolates) (*P* = 0.001). More number of *Corynebacterium* species was isolated from eyes with blepharitis and conjunctivitis [71%; 147 of 207 total *Corynebacterium* isolates (74 isolates from blepharitis and 73 from conjunctivitis)] than any other ocular infection (29%; 60 of 207) (*P* = 0.001). Ps. aeruginosa was isolated at a higher frequency from eyes with dacryocystitis and keratitis (66.5%; 145 of 218) (*P* = 0.001). The recovery of *Moraxella lacunata* (54.17%; 26 of 48 total *M. lacunata* isolates) (*P* = 0.001) and *M. catarrhalis* (63.83%; 60 of 94 total *M. catarrhalis* isolates) (*P* = 0.001) was significantly more in number from eyes with blepharitis and dacryocystitis, respectively, than from eyes with any other ocular infections. Larger numbers of *Nocardia asteroids* (49.06%; 26 of 53 total *Nocardia* isolates) were isolated from corneal ulcers than from any other ocular infections (50.9%; 27 of 53). *Haemophilus* species were isolated from larger number of ocular samples obtained from eyes with dacryocystitis and conjunctivitis [73.2%; 112 (73 from dacryocystitis and 39 from conjunctivitis) of 153 total *Haemophilus* isolates] than from samples with other ocular infections (33.33%; 51 of 153) (*P* = 0.001).

Overall, large numbers of bacterial isolates were susceptible to gatifloxacin (93.68%; 326 of 348) [Table 6]. The highest percentage of gram-positive organisms was susceptible to moxifloxacin (99.1%; 226 of 228), followed by vancomycin (97.93%; 1750 of 1787), gatifloxacin (93.86%; 214 of 228), cefazolin (91.77%; 1640 of 1787) and chloramphenicol (88.86%; 1588 of 1787). The gram-negative organisms were susceptible in highest percentage to amikacin (93.51%; 721 of 771), followed by gatifloxacin (92.66%; 101 of 109), ofloxacin (88.72%; 684 of 771) and ciprofloxacin (86.64%; 668 of 771). Amikacin (100%) and vancomycin (100%) showed highest efficacy against *Nocardia* spp.

**Table 6:** *In vitro* antibacterial susceptibility pattern of bacterial isolates recovered from ocular specimens obtained from (n = 2611) eyes with bacterial ocular infections

	Amikacin	Tobramycin	Gentamicin	Cefaolzin	Cephotaxime	Cetazidime	Norfloxacin	Ciprofloxacin	Ofloxacin	Gatifloxacin	Moxifloxacin	Chloramphenicol	Vancomycin
Gram-positive cocci	52 (810/1553)	44 (686/1553)	48 (740/1553)	92 (1426/1553)	83 (1293/1553)	63 (984/1553)	81 (1257/1553)	82 (1273/1553)	87 (1352/1553)	94 (206/220)	99 (218/220)	95 (1474/1553)	99 (1539/1553)
*Sta. aureus*	99 (691/697)	79 (551/697)	80 (555/697)	89 (619/697)	80 (555/697)	40 (277/697)	87 (606/697)	88 (610/697)	89 (617/697)	96 (101/105)	98 (103/105)	99 (690/697)	100 (697/697)
*CoNS*	73 (119/162)	33 (53/162)	41 (66/162)	75 (122/162)	41 (67/162)	22 (36/162)	41 (67/162)	41 (67/162)	43 (69/162)	95 (20/21)	100 (21/21)	70 (113/162)	100 (162/162)
*Str. pneumoniae*	0 (0/578)	12 (69/578)	17 (99/578)	99 (573/578)	97 (561/578)	97 (561/578)	84 (485/578)	85 (492/578)	96 (554/578)	92 (73/79)	100 (79/79)	97 (561/578)	99 (570/578)
*S. pyogenes*	0 (0/42)	10 (4/42)	17 (7/42)	100 (42/42)	95 (40/42)	95 (40/42)	83 (35/42)	91 (38/42)	95 (40/42)	80 (4/5)	100 (5/5)	95 (40/42)	95 (40/42)
*S. viridans*	0 (0/74)	12 (9/74)	18 (13/74)	99 (73/74)	95 (70/74)	95 (70/74)	87 (64/74)	89 (66/74)	97 (72/74)	80 (8/10)	100 (10/10)	95 (70/74)	95 (70/74)
Gram-positive bacilli	90 (211/234)	58 (136/234)	49 (114/234)	92 (214/234)	86 (202/234)	86 (202/234)	43 (101/234)	61 (142/234)	90 (211/234)	100 (8/8)	100 (8/8)	49 (114/234)	90 (211/234)
*Bacillus* spp	92 (25/27)	59 (16/27)	48 (13/27)	93 (25/27)	48 (13/27)	48 (13/27)	37 (10/27)	48 (13/27)	92.6 (25/27)	100 (4/4)	100 (4/4)	48 (13/27)	92.6 (25/27)
*Corynebacterium* spp.	90 (186/207)	58 (120/207)	49 (101/207)	91 (189/207)	91 (189/207)	91 (189/207)	44 (91/207)	62 (129/207)	90 (186/207)	100 (4/4)	100 (4/4)	49 (101/207)	90 (186/207)
Gram-negative cocci	99 (168/169)	82 (138/169)	81 (136/169)	9 (15/169)	86 (146/169)	91 (154/169)	94 (159/169)	94 (159/169)	99 (168/169)	95 (19/20)	90 (18/20)	88 (149/169)	7 (12/169)
*M. lacunata*	100 (48/48)	96 (46/48)	96 (46/48)	19 (9/48)	83 (40/48)	92 (44/48)	96 (46/48)	96 (46/48)	98 (47/48)	100 (7/7)	86 (6/7)	96 (46/48)	19 (9/48)
*Ne. gonorrhea*	100 (11/11)	73 (8/11)	73 (8/11)	0 (0/11)	82 (9/11)	82 (9/11)	91 (10/11)	91 (10/11)	100 (11/11)	100 (2/2)	100 (2/2)	73 (8/11)	9 (1/11)
*Ne. meningitides*	100 (3/3)	67 (2/3)	67 (2/3)	0 (0/3)	100 (3/3)	100 (3/3)	100 (3/3)	100 (3/3)	100 (3/3)	not done	not done	100 (3/3)	0 (0/3)
*M. catarrhalis*	98.9 (93/94)	77 (72/94)	75 (70/94)	6 (6/94)	87 (82/94)	94 (88/94)	94 (88/94)	94 (88/94)	100 (94/94)	91 (10/11)	91 (10/11)	87 (82/94)	2 (2/94)
*Acinetobacter* spp.	100 (13/13)	77 (10/13)	77 (10/13)	0 (0/13)	92 (12/13)	77 (10/13)	92 (12/13)	92 (12/13)	100 (13/13)	not done	not done	77 (10/13)	0 (0/13)
Aerobic actinomycetes	100 (53/53)	47 (25/53)	47 (25/53)	0 (0/53)	76 (40/53)	13 (7/53)	47 (25/53)	62 (33/53)	76 (40/53)	100 (11/11)	91 (10/11)	25 (13/53)	100 (53/53)
*No. asteroides*	100 (53/53)	47 (25/53)	47 (25/53)	0 (0/53)	76 (40/53)	13 (7/53)	47 (25/53)	62 (33/53)	75.5 (40/53)	100 (11/11)	90.9 (10/11)	25 (13/53)	100 (53/53)
Gram-negative bacilli	92 (553/602)	43 (258/602)	45 (268/602)	4 (24/602)	62 (372/602)	71 (430/602)	79 (476/602)	85 (509/602)	86 (516/602)	92 (82/89)	79 (70/89)	60 (360/602)	0.66 (4/602)
*Ps. aeruginosa*	88 (191/218)	30 (66/218)	33 (71/218)	0 (0/218)	64 (140/218)	80 (180/218)	82 (179/218)	85 (186/218)	87 (189/218)	88 (29/33)	79 (26/33)	60 (131/218)	0 (0/218)
*Es. coli*	96 (23/24)	13 (3/24)	13 (3/24)	0 (0/24)	21 (5/24)	25 (6/24)	33 (8/24)	33 (8/24)	33 (8/24)	67 (2/3)	33 (1/3)	21 (5/24)	0 (0/24)
*H. influenzae*	88 (79/90)	79 (71/90)	81 (73/90)	16 (15/90)	61 (55/90)	66 (59/90)	83 (75/90)	86 (77/90)	88 (79/90)	92 (11/12)	100 (12/12)	61 (55/90)	0 (0/90)
*H. parainfluenzae*	98 (47/48)	83 (40/48)	88 (42/48)	10 (5/48)	75 (36/48)	79 (38/48)	85 (41/48)	94 (45/48)	98 (47/48)	100 (12/12)	100 (12/12)	63 (30/48)	0 (0/48)
*H. aegypticus*	100 (15/15)	87 (13/15)	87 (13/15)	7 (1/15)	80 (12/15)	80 (12/15)	87 (13/15)	100 (15/15)	100 (15/15)	100 (2/2)	100 (2/2)	80 (12/15)	0 (0/15)
*En. agglomerans*	100(16/16)	25 (4/16)	31 (5/16)	0 (0/16)	50 (8/16)	63 (10/16)	88 (14/16)	94 (15/16)	94 (15/16)	100 (2/2)	100 (2/2)	63 (10/16)	0 (0/16)
*En. aerogenes*	96 (47/49)	27 (13/49)	23 (11/49)	0 (0/49)	61 (30/49)	67 (33/49)	78 (38/49)	84 (41/49)	84 (41/49)	100 (7/7)	71 (5/7)	62 (30/49)	0 (0/49)
*En. cloacae*	100 (5/5)	20 (1/5)	20 (1/5)	0 (0/5)	40 (2/5)	60 (3/5)	80 (4/5)	100 (5/5)	100 (5/5)	not done	not done	60 (3/5)	0 (0/5)
*K. ozaenae*	100 (9/9)	44 (4/9)	44 (4/9)	0 (0/9)	78 (7/9)	78 (7/9)	78 (7/9)	89 (8/9)	89 (8/9)	not done	not done	78 (7/9)	0 (0/9)
*K. pneumoniae*	92 (70/76)	28 (21/76)	30 (23/76)	0 (0/76)	58 (44/76)	63 (48/76)	70 (53/76)	80 (61/76)	80 (61/76)	91 (10/11)	55 (6/11)	58 (44/76)	0 (0/76)
*Ci. diversus*	100 (10/10)	40 (4/10)	40 (4/10)	0 (0/10)	70 (7/10)	70 (7/10)	90 (9/10)	90 (9/10)	90 (9/10)	100 (1/1)	100 (1/1)	70 (7/10)	10 (1/10)
*Ci. freundic*	100 (7/7)	57 (4/7)	57 (4/7)	14(1/7)	71 (5/7)	71 (5/7)	86 (6/7)	100 (7/7)	100 (7/7)	100 (1/1)	100 (1/1)	72 (5/7)	14(1/7)
*Al. faecalis*	100 (9/9)	44 (4/9)	44 (4/9)	0 (0/9)	78 (7/9)	78 (7/9)	78 (7/9)	89 (8/9)	89 (8/9)	100 (1/1)	0 (0/1)	78 (7/9)	0 (0/9)
*Pr. mirabilis*	91 (10/11)	27 (3/11)	27 (3/11)	0 (0/11)	36 (4/11)	36 (4/11)	81 (9/11)	81 (9/11)	82 (9/11)	100 (2/2)	0 (0/2)	36 (4/11)	0 (0/11)
*Pr. vulgaris*	100 (5/5)	20 (1/5)	20 (1/5)	0 (0/5)	40 (2/5)	60 (3/5)	80 (4/5)	100 (5/5)	100 (5/5)	not done	not done	40 (2/5)	0 (0/5)
*Se. marcesens*	100 (10/10)	60 (6/10)	60 (6/10)	20 (2/10)	80 (8/10)	80 (8/10)	90 (9/10)	100 (10/10)	100 (10/10)	100 (2/2)	100 (2/2)	80 (8/10)	20 (2/10)
Total	68.74 (1795/2611)	47.6 (1243/2611)	49.14 (1283/2611)	64.3 (1679/2611)	78.63 (2053/2611)	68.06 (1777/2611)	77.29 (2018/2611)	81.04 (2116/2611)	87.59 (2287/2611)	93.68 (326/348)	93.1 (324/348)	80.81 (2110/2611)	70.43 (1839/2611)

## Discussion

A combination of mechanical, anatomic, immunologic and microbiologic factors prevents ocular infections and do not allow the survival of pathogenic species in the eye.[18,19] However, in certain circumstances, they gain access to the eye and cause a variety of infections. Prompt and specific therapy can be instituted if the microbes can be isolated and their susceptibility to the antimicrobials is known. However, the ability to isolate the causative organism depends on a variety of factors including the amount of inoculum,[20] the site from which it is taken, the media used for culture (whether enriched media are used or not)[21] and also on the empirical treatment received before collection of the samples.[22] Hence, the culture-positivity varies from center to center. In this study, the overall culture-positivity was 69.8%. We found the highest rate of culture-positivity among the samples collected from eyelid infections probably due to two reasons, that is, being the outermost defense mechanism it harbors a large number of microorganisms and the amount of inoculum is also sufficient to inoculate the various media.

In this study, bacteria (58.8%) were the most common pathogens and were involved in infections of all the tissues of the eye, whereas fungi (10.3%) caused keratitis and endophtalmitis and *Acanthamoeba* (0.34%) caused only keratitis. The most common bacteria isolated from ocular specimens were *Sta. aureus* (26.69%) followed by *Str. pneumoniae* (22.14%). *Sta. aureus* caused infections of the eyelids (52.57%), conjunctiva (40.45%) and canaliculus (33.85%), whereas *Str. pneumoniae* caused lacrimal sac (31.35%) and corneal infections (37%) and CoNS, postoperative (68.27%) and post-traumatic endophthalmitis (33%). Though *Staphylococci* and *Streptococci* along with other bacteria like *Corynebacterium, Haemophilus, Moraxella* and *Neisseria* are part of the normal flora of the conjunctiva, under appropriate conditions they cause infections.[2,23,24] *Sta. aureus*is commonly involved in primary pyoderma and acts as a secondary invader on diseased skin. It produces coagulase, a factor capable of clotting the plasma which may play a role in the development of staphylococcal abscess by producing local fibrin thrombi that protect organisms and concentrate toxic factors.[25] CoNS elaborate a surface slime that facilitates adherence to the surface and may play a role in the pathogenesis of endophthalmitis. The surface slime protects the organism from phagocytosis and the action of antimicrobial agents. CoNS, especially *Staphylococcus epidermidis* is the commonest cause for postoperative endophthalmitis.[6,7] Being a normal inhabitant of the upper respiratory tract, *Str. pneumoniae* is frequently found in the lacrimal apparatus and conjunctiva.[2,3] Any minor corneal epithelial disruption facilitates invasion of the bacteria, hence causing corneal ulcer.

Among gram-negative bacilli, the most common pathogen was *Pseudomonas* spp. (8.4%), followed by *Haemophilus* spp. (5.9%), *Klebsiella* spp. (3.2%) and *Enterobacter* spp. (2.7%). Prevalence of *Pseudomonas* spp. was more in keratitis (34.9%; 76 of 218) and dacrocystitis (31.7%; 69 of 218), *Haemophilus* spp. in dacryocystitis (47.7%; 73 of 153) and conjunctivitis (25.5%; 39 of 153), *Klebsiella* spp. in dacryocystitis (62.4%; 53 of 85) and *Enterobcater* spp. in both keratitis (35.7%; 25 of 70) and dacryocystitis (34.3%; 24 of 70). *Pseudomonas* keratitis has been attributed to the action of proteases and glycocalyx that allow the organisms that adhere to the host cells forming micro colonies that resist phagocytosis.[26] Natural pathogenicity of *Haemophilus* appears to be directly related to the capsule formation which renders resistance to complement-mediated immunity.[27] The gram-negative bacilli, *Klebsiella* spp., *Enterobacter* spp., *Citrobacter* spp., *Proteus* spp., *Serratia* spp. etc., are found in soil and sewage and are opportunistic pathogens causing conjunctivitis, keratitis, dacryocystitis, orbital cellulitis and endophthalmitis when the host defenses are low.[4,5]

Among the gram-negative coccobacilli, the predominant isolate, *M. catarrhalis* demonstrated 3.6% of incidence and was more frequently present in dacryocystitis (63.8%; 60 of 94), whereas *M. lacunata* was prevalent more in blepharitis (54.2%; 26 of 48), *Neisseria* in conjunctivitis (57%; 8 of 14). *M. cararrhalis*, a constant inhabitant of the respiratory tract, tends to cause dacryocystitis and less frequently meibomitis, conjunctivitis, keratitis and rarely postoperative endophthalmitis.[28] *M. lacunata* is commonly found in hot and dry areas of the world and causes angular conjunctivitis in alcoholics and debilitated patients.[29] *Neisseria* spp. infects mucosa of genitourinary tract and conjunctiva of neonates, adolescents and adults.[28]

The filamentous bacteria, *Nocardia*, accounted for 2% of the incidence and its prevalence was 49% in keratitis and 20.7% in canaliculitis. In comparison, the incidence of gram-positive bacilli was 9%, of which *Corynebacterium* spp. accounted a higher rate of prevalence in blepharitis (35.7%; 74 of 207) and conjunctivitis (35.3%; 73 of 207), whereas *Bacillus* species was present in conjunctivitis (40.7%; 11 of 27) and in post-traumatic endophthalmitis (26%; 7 of 27). *Nocardia* infection usually occurs following trauma with objects contaminated with soil, and there have been sporadic reports of conjunctivitis, dacryocystitis, canaliculitis, scleritis, keratits, episcleral granuloma and endophthalmitis.[30] *Corynebacterium* spp. are almost constant saprophytes in the conjunctiva, however, *Corynebacterium diphtheriae* causes severe membranous conjunctivitis associated with pharyngeal diphtheria.[31] *Bacillus* spp. are ubiquitous in nature and are known to cause severe endophthalmitis following penetrating injury with metallic or vegetative foreign bodies and also by endogenous spread in drug abusers.[32]

Resistance and sensitivity based on *in vitro* testing may not reflect true clinical resistance and response to an antibiotic because of the host factors and penetration of the drug. In this study, moxifloxacin and vancomycin revealed a higher efficacy against gram-positive isolates compared with other antibacterial agents. Vancomycin is a glycopeptide; it inhibits early stages in cell wall mucopeptide synthesis and it exhibited greatest potency against ocular gram-positive isolates. Moxifloxacin was specifically developed with methoxy group in the C-8 position and bicyclic side-chain in the C-7 position, which was specifically engineered to increase the potency and further inhibit bacterial resistance by hindering the cell’s efflux pump mechanism, increasing the drug’s length of stay within bacterial cells. Recent studies have also shown the excellent gram-positive coverage of moxifloxacin in ocular infections.[33] However, moxifloxacin has incomplete coverage against gram-negative isolates. We found greatest coverage of gatifloxacin and amikacin against gram-negative isolates. Ciprofloxacin and ofloxacin were introduced earlier and have been widely used since 1990, whereas gatifloxacin’s usage has started in recent years. In addition to methoxy side chain at the C-8 position, gatifloxacin carries a methyl group on the piperazinyl ring. There was a slight decrease in all pathogens’ susceptibilities to ciprofloxacin and ofloxacin, with a subsequent increase in the efficacy of gatifloxacin.[33] The relationship between antibiotic use and resistance is complex. Improper selection of antibiotics, inadequate dosing and poor compliance to therapy may play as important a role in increasing resistance as their overuse. This report documents the prevalence of bacterial species causing ocular infections in South India. The information provided in this article would aid the clinician in formulating rationale-based decisions in the antibiotic treatment of bacterial ocular infections that cause major public health problems.
